# The Manchester Color Wheel: validation in secondary school pupils

**DOI:** 10.1186/1471-2288-12-136

**Published:** 2012-09-05

**Authors:** Helen R Carruthers, Linda Magee, Susan Osborne, Linda K Hall, Peter J Whorwell

**Affiliations:** 1 Department of Medical Illustration, University Hospital of South Manchester NHS Foundation Trust, Manchester, UK; 2 Manchester Academic Health Science Centre, University of Manchester, Manchester, UK; 3 Sale Grammar School, Manchester, UK; 4 Centre for Gastrointestinal Sciences, University of Manchester, UK, Education and Research Centre, University Hospital of South Manchester NHS Foundation Trust, Manchester, M23 9LT, UK

**Keywords:** Color perception, Manchester Color Wheel, Adolescents

## Abstract

**Background:**

As part of our research programme into facilitating improved ways of communicating with patients, especially about more sensitive clinical issues, we have been investigating whether there are any non-verbal methods that might aid this process. One such approach is to ask patients to choose a color in response to a particular question, for instance about health or psychological status, and for this purpose we developed the Manchester Color Wheel (MCW). This instrument consists of positive, neutral and negative colors and its validation in normal adults and those with anxiety or depression showed that it is responsive to change and reproducible. It also has the capacity to identify a positive frame of mind. We concluded that it might be a particularly useful instrument in adolescents and therefore this study aimed to validate it in a secondary school.

**Methods:**

620 pupils (aged 11–17 years, mean age 14.0 years, 298 (48.1%) males, 322 (51.9%) females) at Sale Grammar School in Greater Manchester were asked to relate their mood to a MCW color and also complete the Hospital Anxiety Depression (HAD) questionnaire. To give these pupils an experience in science, 197 were divided into four subgroups for an ‘experiment’ to ascertain whether, compared to controls, a change in mood color choice could be induced by participation in sport, music or art activities.

**Results:**

Although mood color and HAD depression score are unlikely to be measuring exactly the same psychological state, a negative mood color was chosen by 62.5% of HAD depressed compared to only 14.5% of HAD normal pupils (p < 0.001). In contrast, a positive mood color was chosen by 48.9% of normal and only 18.8% of depressed pupils (p < 0.001). In the ‘experiment’, compared to controls, all activities resulted in an increased choice of positive mood colors which reached significance for sport and music.

**Conclusion:**

This study confirms the potential utility of the MCW to rapidly and easily assess a variety of health issues in large populations, including adolescents. Some of our results should also be of interest to educationalists.

## Background

Communication between a patient and their doctor during a consultation can sometimes be inhibited by factors such as the embarrassing nature of their complaint, difficulty describing their symptoms, failure to understand questions or problems over language. In order to try and overcome some of these issues we have been exploring the utility of imagery and color in helping patients to convey how they feel [1-4]. Our results to date suggest that presenting patients with visual images depicting a variety of symptoms, such as different types of pain, enables them to more easily express themselves [1,2]. Furthermore, we have found that how individuals respond to questions in terms of color can also have a range of applications [3].

**Figure 1:**
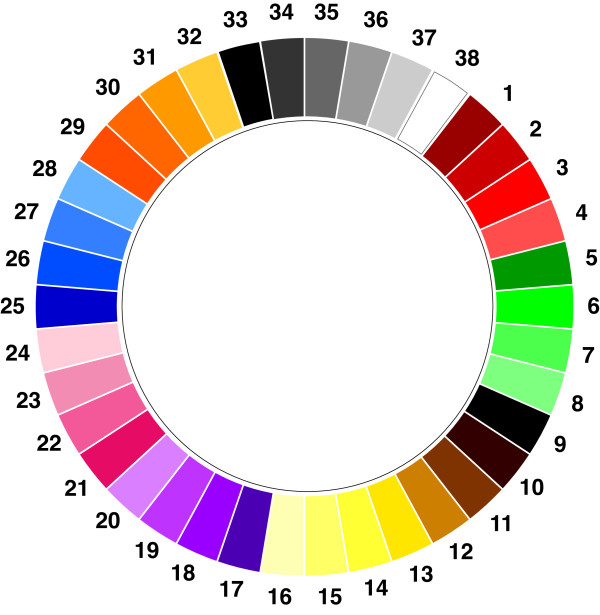
The ‘Manchester Color Wheel’ (3).

For the latter purpose we developed the ‘Manchester Color Wheel’ (MCW) as a simple way of presenting 38 different shades of color to an individual, so that their color choice could be related to their health or psychological status (Figure 1) [3]. Validation in healthy individuals as well as those with affective disorders facilitated the categorisation of colors into positive, neutral and negative shades where it was found that the shade of a particular color, for instance light or dark, was just as important as the actual color itself in determining how an individual reacted to it. Our results demonstrated that by asking an individual to relate their mood to a color, the choice of a negative shade was linked to depression and, to a lesser extent, anxiety. Consequently, we concluded that although mood color choice and depression score are probably not measuring exactly the same phenomena, the MCW might act as a rapid screening tool for detecting the possibility of a low mood state, which could then be further investigated by the use of more detailed questionnaires. In further studies we have also shown, that when patients are asked to relate their disease rather than their mood to a color, they associate it with a negative shade irrespective of whether it gets better or not, whereas their mood color becomes positive when symptoms improve [4]. Another useful application of the MCW is that, in contrast to standard questionnaires to identify anxiety or depression, it can be used in a more constructive way to detect a positive frame of mind.

So far, the MCW has only been used in studies in adults in the UK and further validation in adolescents, as well as in different cultures, is necessary. In September 2011, a communications exercise called the ‘Great Manchester Health Experiment’ was undertaken to try and engage the general public in the importance of health research and health issues under the auspices of the Manchester Academic Health Science Centre (MAHSC) which is a partnership between the University of Manchester and six NHS Hospitals (Appendix 1) in the Manchester conurbation. The basis of the ‘Experiment’ was to use the MCW to measure the ‘mood of Manchester’ in the morning and afternoon on a particular day and to encourage the public to engage in activities associated with wellbeing in the intervening period. This also presented us with a golden opportunity to involve a school in a more ‘scientific’ component of the project. Consequently, the pupils and staff of a local grammar school were asked to participate in an exercise to help validate the MCW in a large group of adolescents as well as investigating whether the mood of the school could be altered by a variety of different activities aimed at influencing how they might feel. In an attempt to further encourage interest in health research, the production of this paper was used as a working example of how to analyse data and compose a scientific publication with the involvement of the school.

In order to aid the validation process of the MCW in this school population we needed to identify a comparator group of pupils where color choice might be distorted in some way. In our previous adult study we found that individuals with anxiety or depression exhibited a different mood color choice distribution to normal people and it was therefore decided to screen the participants of the current study for these two problems for the purpose of identifying such a subgroup.

## Methods

**Figure 2:**
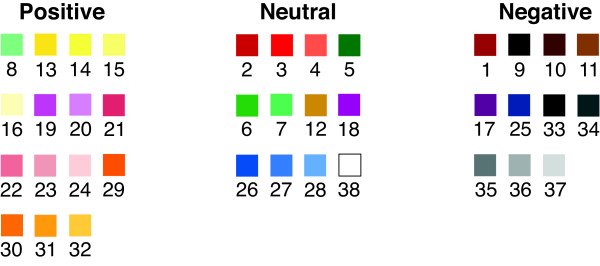
Classification of colors from the MCW into positive, neutral and negative shades (3).

For the purpose of this investigation, we used a permutation from our previous study [3] which divided the colors from the MCW into approximately equal numbers of positive, neutral and negative shades (Figure 2). For clarity in the results, the colors are always grouped into positive, neutral and negative shades rather than by their numerical position on the MCW (Figure 1). For the purposes of analysis and showing results each color on the MCW is given a number but this is removed when the MCW is shown to participants in order to prevent any possible confusion. In our validation study, we have advised that if the MCW is for any reason shown to the same individual on multiple occasions that it might be worth considering rotating it in case the position of colors becomes memorised. However, in this study the MCW was only shown twice to participants, with an interval of six hours between questionnaires, and it was therefore felt that rotation was unnecessary. Furthermore, participants were given no indication of which color is considered positive, neutral or negative.

### Study 1 – Application of the Manchester Color Wheel in pupils grouped, according to their Hospital Anxiety and Depression Scores, into normal, anxious or depressed individuals

As part of the Great Manchester Health Experiment, 663 pupils from Sale Grammar School in Manchester (aged 11–17, mean age 14.0 years, 319 (48.1%) males, 344 (51.9%) females) were recruited into this study where they were asked to complete a Color Questionnaire using the MCW. The following questions were asked: 1. *Do you associate a color with your mood?* 2. *Irrespective of your answer to Question 1, please put a cross by the color that best matches your current mood.* 3. *Is there a reason why you have chosen this color?* 4. *Do you suffer from any medical condition requiring ongoing treatment?* All participants were also asked to complete the Hospital Anxiety and Depression (HAD) Scale [5] so that the data of those with significant anxiety and depression (a score of 10 or greater for each domain) could be compared with those scoring normally on the HAD Scale. Pupils suffering from any medical condition requiring regular treatment were excluded from the study as well as any individuals with color vision deficiency (color blindness). 34 subjects were suffering from a medical condition and 9 failed to complete the questionnaire satisfactorily and were therefore excluded from the study. This left 620 pupils (aged 11–17 years, mean age 14.0 years, 298 (48.1%) males, 322 (51.9%) females) for the analysis. No details which could lead to the identification of any of the participants either directly or indirectly, such as name or date of birth, were recorded with only age and sex being documented.

Of these 620 pupils, 462 (74.5%) participants were classified as HAD normal and were aged 11–17 years (mean age 13.9 years) with 232 (50.2%) being male and 230 (49.8%) being female. 122 (19.7%) were classified as HAD anxious participants (aged 11–17 years, mean age 14.5 years, 46 (37.7%) males, 76 (62.3%) females) and 36 (5.8%) subjects were classified as HAD depressed (aged 11–17 years, mean age 14.5 years, 20 (55.6%) males, 16 (44.4%) females).

### Study 2 – Short term mood change in response to differing activities

As a further extension of the Great Manchester Health Experiment, a subgroup of 278 pupils from Sale Grammar School (aged 14–17, mean age 15.3 years, 124 (44.4%) males, 154 (55.4%) females) were asked to take part in a study, utilising the MCW, to assess whether a short term improvement in mood could be induced by a variety of different activities. No details which could lead to the identification of any of the participants either directly or indirectly, such as name or date of birth, were recorded with only age and sex being documented. At 9.00 am, they were asked to complete exactly the same questionnaires as those participating in Study 1 including the Hospital Anxiety and Depression (HAD) Scale [5] and then divided into four groups depending on the class to which they belonged. The first group continued with their normal school curriculum and acted as controls whilst participants in the other three groups were assigned to a particular activity designed to improve mood. A second Color Questionnaire was then completed at 3.00 pm and simply asked, *“Please put a cross by the color that best matches your current mood”.* Pupils suffering from any medical condition requiring regular treatment were excluded from the study as well as any individuals with color vision deficiency (color blindness). 14 subjects were suffering from a medical condition and 67 failed to complete one or other of the questionnaires and were therefore excluded from the study leaving 197 pupils (aged 14–17 years, mean age 15.05 years, 90 (45.7%) males, 107 (54.3%) females) who were assigned into the following four categories:

### Control group

25 participants (aged 15–17 years, mean age 15.48 years, 10 (40.0%) males, 15 (60.0%) females) carried on with their normal school timetable. Alternative control activities such as letting pupils have the day off were considered but it was felt that it would have been impossible to monitor what they were doing and that it would have also presented logistical problems in terms of ensuring the return of questionnaires.

### Sport group

67 participants (aged 14–16 years, mean age 15.04 years, 37 (55.2%) males, 30 (44.8%) females) took part in various sporting activities such as dodge ball for one hour.

### Music group

82 participants (aged 15 years, mean age 15.00 years, 41 (50.0%) males, 41 (50.0%) females) listened to live music being played, ranging from classical to rock music. One student played the piano and included chart songs in his repertoire.

### Art group

23 participants (aged 14–17 years, mean age 14.74 years, 2 (8.7%) males, 21 (91.3%) females) worked in groups to construct sculptures. The pupils were set the challenge to produce a piece of artwork using wood and other art materials.

### Statistical analysis

The percentage of respondents selecting an individual color in terms of it being their ‘mood’ color was calculated.

The statistical package SPSS 15 (SPSS Inc., Chicago, Illinois, USA) was used for analysing the data. The Pearson Chi-squared test was used to assess the differences between the three volunteer groups (HAD normal, HAD anxious and HAD depressed) and mood color choice. The McNemar-Bowker Test was used to compare mood color choice at 9.00 am and 3.00 pm in each of the four intervention groups.

### Ethical review

The study was submitted to the Northwest 6 Research Ethics Committee – Greater Manchester South for consideration. It was decided that the project fell outside the Governance Arrangements for NHS Research Ethics Committees Section (GAfREC) and therefore did not require formal ethical review. Parental and guardian consent was sought and obtained by the school prior to the commencement of the study.

## Results

### Study 1 – application of the Manchester Color Wheel in pupils grouped, according to their Hospital Anxiety and Depression Scores, into normal, anxious or depressed individuals

**Figure 3:**
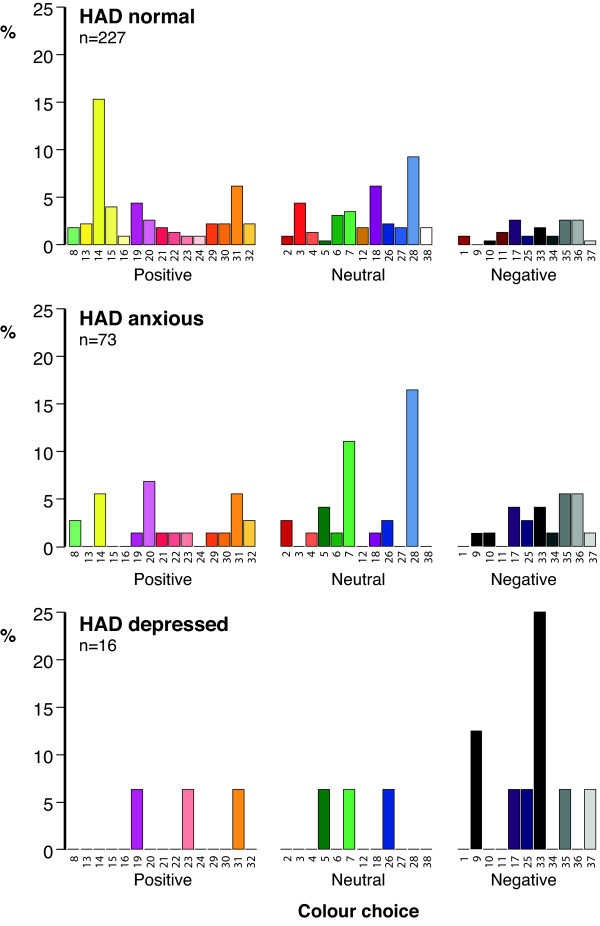
**Distribution of ‘spontaneous’ mood color choice in HAD normal, anxious and depressed adolescents.** 227 (49.1%) HAD normal subjects, 73 (59.8%) HAD anxious and 16 (44.4%) HAD depressed pupils related a color to their mood. Adolescents not choosing a color to describe their mood have been omitted from the figure.

In response to the question *“Do you associate a color with your mood*”, 316 (51.0%) of the 620 participants claimed to associate a color with their mood and the distribution of their color choices are shown in Figure 3. As can be seen, 227 (49.1%) of the 462 HAD normal participants associated a color with their mood and the most popular color was ‘Yellow 14’ which was chosen by 35 (15.4%) individuals. The reasons they gave for choosing this color were because they were feeling ‘happy’, ‘in a good mood’, ‘very happy, excited and proud’, ‘because it’s like sunshine and I’m happy’, ‘bright and cheerful’ and ‘it’s my birthday tomorrow and I’m happy’. 73 (59.8%) of the 122 HAD anxious participants associated a color with their mood, with 12 (16.4%) choosing ‘Blue 28’ giving reasons such as, ‘I’m calm but also cold’, ‘I’m a bit sad’, ‘I’m sad at the moment’ and ‘peaceful’. 16 (44.4%) of the 36 HAD depressed participants associated their mood with a color with 4 (25%) choosing ‘Black 33’ giving reasons such as, ‘I don’t have many friends’, ‘feeling sad and slightly depressed’, ‘tired’ and ‘not very happy’. Tiredness seemed to be a rather common complaint and was associated with negative colors even in the HAD normal population.

Table 1 compares the positive, neutral and negative color choice in those HAD normal, HAD anxious and HAD depressed participants choosing a color to describe their mood. As can be seen, there were highly significant differences between color choice and the three participant groups (Chi-square (4) = 27.893; p < 0.001). HAD depressed individuals showed a striking preference for negative colors choosing relatively few positive shades when compared to HAD normal individuals. HAD anxious individuals gave results intermediate to the HAD depressed and HAD normal subjects.

**Table 1:** Comparison of ‘spontaneous’ mood color choice in the HAD normal, HAD anxious and HAD depressed adolescents

**Color choice**	**Volunteer group**			**p value**
	**HAD normal (n = 227) *No. (%) *Respondents**	**HAD anxious (n = 73) *No. (%) *Respondents**	**HAD depressed (n = 16) *No. (%) *Respondents**	
Positive	111 (48.9%)	23 (31.5%)	3 (18.8%)	Chi-square (4) = 27.893; p < 0.001
Neutral	83 (36.6%)	30 (41.1%)	3 (18.8%)	
Negative	33 (14.5%)	20 (27.4%)	10 (62.5%)	

**Figure 4:**
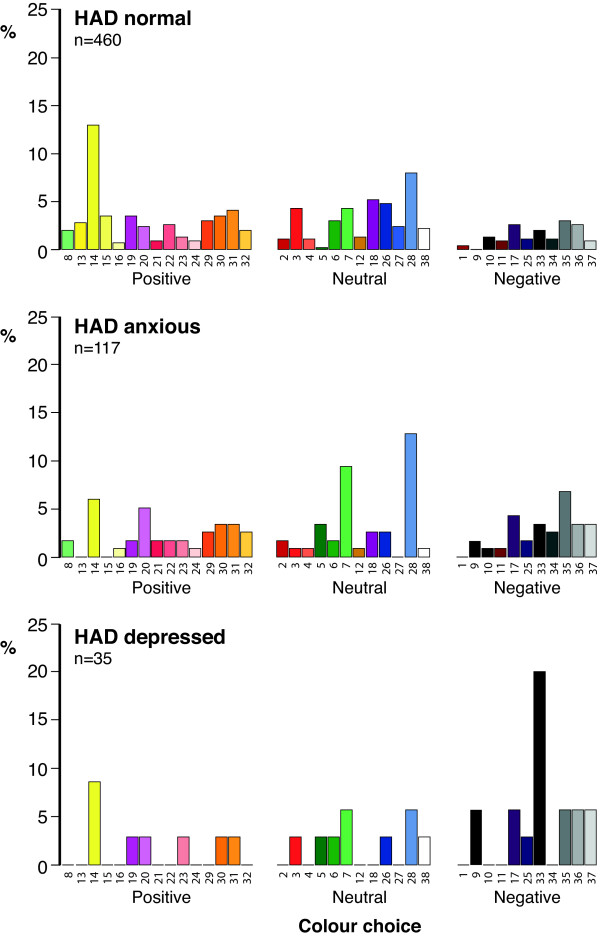
**Distribution of ‘forced’ mood color choice in HAD normal, anxious and depressed adolescents.** 460 (99.6%) HAD normal subjects, 117 (95.9%) HAD anxious and 35 (97.2%) HAD depressed pupils related color to their mood. Adolescents not choosing a color to describe their mood have been omitted from the figure.

When the participants were then asked *“Irrespective of your answer to Question 1, please put a cross by the color that best matches your current mood”,* 612 (98.7%) of the 620 participants now related a color to their mood and the distribution of their color choices are shown in Figure 4. As can be seen, 460 (99.6%) HAD normal participants responded to this question with 60 (13%) choosing ‘Yellow 14’. 117 (95.9%) HAD anxious participants responded to the question with ‘Blue 28’ being chosen by 15 (12.3%) participants. 35 (97.2%) HAD depressed participants responded to the question with 7 (19.4%) choosing ‘Black 33’ to describe their mood.

Table 2 compares the positive, neutral and negative color choice in those HAD normal, HAD anxious and HAD depressed volunteers now choosing a color to describe their mood. When pupils were encouraged to choose a color irrespective of their response to Question 1, their response was remarkably similar with highly significant differences between color choice and the three volunteer groups (Chi-square (4) = 33.656; p < 0.001). HAD depressed individuals showed a striking preference for negative colors choosing relatively few positive colors when compared to HAD normal individuals. HAD anxious individuals gave results intermediate to the HAD depressed and HAD normal subjects.

**Table 2:** Comparison of ‘forced’ mood color choice in the HAD normal, HAD anxious and HAD depressed adolescents

**Color Choice**	**Volunteer group**			**p value**
	**HAD normal (n = 460) *No. (%) *Respondents**	**HAD anxious (n = 117) *No. (%) *Respondents**	**HAD depressed (n = 35) *No. (%) *Respondents**	
Positive	212 (46.1%)	39 (33.3%)	8 (22.9%)	Chi-square (4) = 33.656; p < 0.001
Neutral	175 (38.0%)	44 (37.6%)	9 (25.7%)	
Negative	73 (15.9%)	34 (29.1%)	18 (51.4%)	

### Study 2 – Short term mood change in response to differing activities

**Figure 5:**
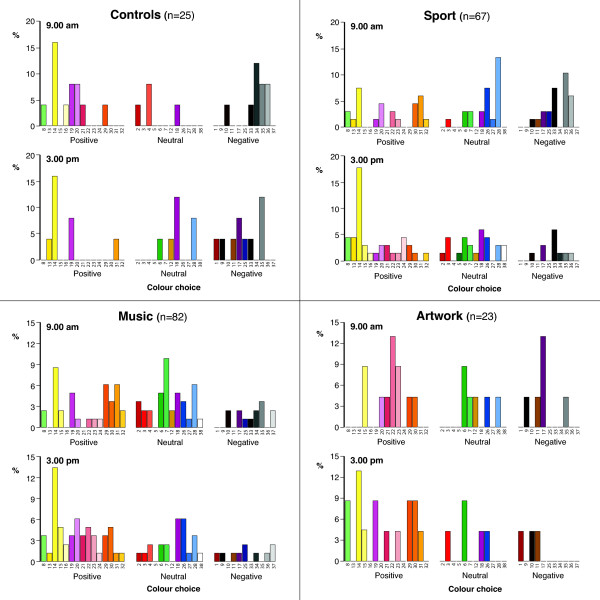
**Overall distribution of mood color choices of the Control, Sport, Music and Art Groups at 9.00 am and 3.00 pm.** Sport, Music and Art activities resulted in more pupils choosing a positive color by the afternoon compared to the morning, whereas in Controls the reverse happened with more participants choosing a negative color by the afternoon. There was an approximately 17% shift towards positive colors with all three interventions which reached significance for Sport (p = 0.047) and Music (p = 0.054).

Figure 5 shows the overall distribution of colors in the four groups at 9.00 am and then at 3.00 pm.

### Control group (n = 25)

The mean HAD anxiety score for this group was 8.8 and HAD depression score was 4.2 at 9.00 am. Table 3 shows that of the 9 individuals who chose a negative color at 9.00 am, 5 remained negative, two converted to a neutral shade and two to a positive color at 3.00 pm. Similarly, of the 12 who chose a positive color at 9.00 am, only 5 remained positive, two changed to a neutral shade and 5 converted to a negative color at 3.00 pm. There were no statistically significant differences in mood change between 9.00 am and 3.00 pm (McNemar-Bowker Test (3) =3.619; p = 0.306).

**Table 3:** The mood color choices of the Control, Sport, Music and Art Groups at 9.00 am and 3.00 pm

**Intervention**	**Mood color**	**Number (%) of respondents at 9.00 am**	**Number (%) of respondents at 3.00 pm**	**p value**
Control (n = 25)	Positive	12 (48.0%)	8 (32.0%)	McNemar-Bowker Test (3) =3.619; p = 0.306
	Neutral	4 (16.0%)	7 (28.0%)	
	Negative	9 (36.0%)	10 (40.0%)	
Sport (n = 67)	Positive	23 (34.3%)	35 (52.2%)	McNemar-Bowker Test (3) =7.965; p = 0.047
	Neutral	22 (32.8%)	22 (32.8%)	
	Negative	22 (32.8%)	10 (14.9%)	
Music (n = 82)	Positive	34 (41.5%)	49 (59.8%)	McNemar-Bowker Test (3) =7.643; p = 0.054
	Neutral	35 (42.7%)	23 (28.0%)	
	Negative	13 (15.9%)	10 (12.2%)	
Art (n = 23)	Positive	11 (47.8%)	15 (65.2%)	McNemar-Bowker Test (3) =1.619; p = 0.655
	Neutral	6 (26.1%)	5 (21.7%)	
	Negative	6 (26.1%)	3 (13.0%)	

### Sport group (n = 67)

The mean HAD anxiety score for this group was 7.9 and HAD depression score was 4.9 at 9.00 am. Table 3 shows that there was a statistically significant mood change in participants between 9.00 am and 3.00 pm with more individuals choosing a positive color to describe their mood following the sporting intervention (McNemar-Bowker Test (3) =7.965; p = 0.047). Of the 22 pupils who chose a negative color at 9.00 am, 10 converted to a positive color, 8 changed to a neutral shade and only 4 remained negative at 3.00 pm. Of the 23 individuals who chose a positive color at 9.00 am, 16 remained positive, 4 converted to a neutral shade and only 3 changed to a negative shade.

### Music group (n = 82)

The mean HAD anxiety score for this group was 7.2 and HAD depression score was 4.2 at 9.00 am. Table 3 shows that there was a statistically significant mood change in participants between 9.00 am and 3.00 pm with more individuals choosing a positive color to describe their mood following the music intervention (McNemar-Bowker Test (3) =7.643; p = 0.054). Of the 13 individuals who chose a negative color at 9.00 am, 6 converted to a positive color at 3.00 pm, 3 changed to a neutral shade and 4 remained negative. Of the 34 individuals who chose a positive color at 9.00 am, 24 remained positive, 9 changed to neutral and only 1 converted to a negative shade.

### Art group (n = 23)

The mean HAD anxiety score for this group was 7.9 and HAD depression score was 3.7 at 9.00 am. Table 3 shows that of the 6 individuals who chose a negative color to describe their mood at 9.00 am, five converted to a positive color and one to a neutral shade at 3.00 pm and of the 11 who chose a positive color at 9.00 am 8 remained positive, one neutral and two converted to a negative color at 3.00 pm. There were no statistically significant differences in mood change between 9.00 am and 3.00 pm (McNemar-Bowker Test (3) =1.619; p = 0.655).

There were no statistically significant differences between any of the groups with respect to mood color choice at 9.00 am (Chi-square (6) = 11.461; p = 0.075) with 34.3%, 41.5%, 47.8% and 48.0% choosing positive colors in the sport, music, art and control groups respectively. In contrast, by 3.00 pm statistically significant differences emerged (Chi-square (6) = 13.494; p = 0.036) with a positive color being chosen in 52.2%, 59.8%, 65.2% and 32.0% of the sport, music, art and control groups respectively. All activities (sport, music and art interactions) resulted in a 17-18% increase in individuals choosing a positive color whereas in controls there was a drop of 16% in those choosing a positive color.

## Discussion

The results of this study suggest that the MCW can be used for research in adolescents as well as adults. The validation process in adults used clinically diagnosed cases of anxiety and depression whereas the present study had to depend on finding chance cases amongst an apparently healthy population of school pupils and consequently, it is likely that in some of these individuals the problem might not have been so clinically significant. Despite this possibility and the fact that the number of affected individuals was rather small, highly significant differences emerged.

### Study 1 – application of the Manchester Color Wheel in pupils grouped, according to their Hospital Anxiety and Depression Scores, into normal, anxious or depressed individuals

**Figure 6:**
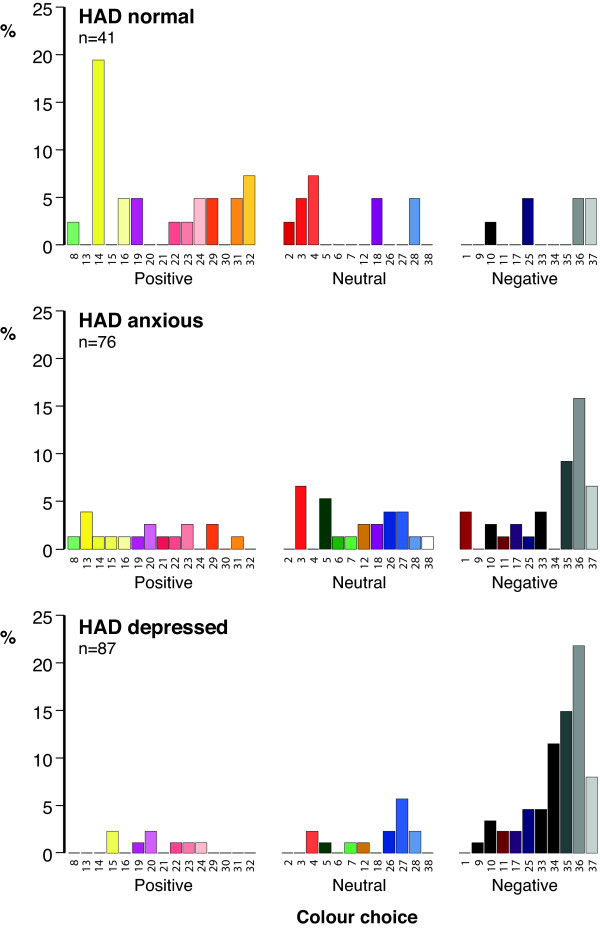
**Distribution of ‘mood’ color choice in HAD normal, anxious and depressed adults.** 41 (39%) HAD normal subjects, 76 (70%) anxious and 87 (79%) depressed volunteers related color to their mood. Volunteers not choosing a color to describe their mood have been omitted from the figure.

The HAD Scale Questionnaire has been validated for use in adolescents [6] and using this instrument 462 (74.5%) pupils were normal, 122 (19.7%) were anxious and 36 (5.8%) depressed. Anxiety was more common in girls whereas there was no difference between the sexes with regard to depression (Chi-square (2) = 6.911; p = 0.032). In response to the question *“Do you associate a color with your mood”*, the percentages choosing a color were 49%, 60% and 44% respectively for normal, anxious and depressed pupils which is in contrast to figures of 39%, 70% and 79% found in the previous study in adults [3]. The fact that more depressed adults chose a color than adolescents is possibly a reflection of the more entrenched nature of their condition compared to the more ‘volatile’ nature of mood in adolescents. Alternatively this observation may be because in the adult study the question was worded somewhat differently: *“With regard to your day-to-day mood over the last few months – do you associate it with a color?”* This question could not be used in the current investigation as we needed a question to capture current mood as, in a subgroup of individuals (Study 2), we were looking for a change over a very short period of time. However, as can be seen from Figures 3, 4 and 6 the distribution of color choice was remarkably similar to what we have previously reported in adults [3] with normal individuals choosing more positive colors and depressed individuals showing a preference for negative colors (Figures 3, 4 and 6) with black being the most favoured negative color in adolescents compared with grey in adults.

One of the problems of asking people if they associate a color with their mood is that not all participants both in this study and the previous one in adults [3] spontaneously respond with a color. For the purposes of the second part of this study we needed as many pupils to choose a mood color as possible and therefore thought it would be useful for validation purposes to ‘force’ participants into making a choice to see whether there were any differences between this group and those ‘spontaneously’ choosing a mood color. As can be seen from Tables 1 and 2 there was very little difference between these groups suggesting that in the future it would be advisable to phrase a question in such a way as to force participants into making a choice. For instance, a near doubling of the identification of people with low mood could be achieved if the MCW was applied using this approach.

The choice of monochromatic shades such as black and grey in individuals with depression is in keeping with the observations of Goodwin and Jamison who stated that depressed patients thought that life was *“colorless”*[7]. Furthermore, Barrick et al. stated that depressed people seemed to be less sensitive to color [8] and De Leo and colleagues claimed that such individuals viewed life ‘*monochromatically’*[9]. Previously, the authors of the present study examined the effect of color choice on response to hypnotherapy treatment in a group of irritable bowel syndrome patients [4]. It was found that depressed patients who responded to hypnotherapy had completely different color choice characteristics to the depressed non-responders. Fewer responders selected a negative color to describe their mood and this was interpreted as indicating that their depression, as identified by the HAD Scale, was not so clinically significant. Consequently, it is possible that the HAD depressed pupils in this study who did not choose a negative color may have some form of more transitory mood change.

Tiredness was quite frequently given as a reason for a negative color choice irrespective of whether a pupil was rated normal, anxious or depressed on the HAD Scale. It is noteworthy that lack of sleep in adolescents has a range of adverse effects on mood as well as motivation and, it has even been suggested, that it can impair the learning process [10,11]. Furthermore, it has been shown that adolescents with late bedtimes are more likely to suffer from depression and that having earlier set bedtimes has a protective effect [12]. The United States National Sleep Foundation has recommended that adolescents should have at least 8.5 hours sleep on a school night in order to maintain normal function the following day [13]. Consequently, it might be of value to enquire about sleep patterns in adolescents reporting negative color choices as this is an issue that is potentially remediable and, if addressed, might lead to better academic performance.

By far the most common positive color chosen by HAD normal pupils was ‘Yellow 14’ and this is exactly the same as we have observed in adults [3]. This is also entirely consistent with the literature on color and mood which indicates that yellow is frequently associated with a positive mood state [14-17]. HAD anxious pupils exhibited a color choice which was intermediate between normal and depressed, which is again similar to that observed in adults. However, the actual colors chosen were different with ‘Blue 28’ being particularly favoured whereas grey was a more common choice in anxious adults. Reasons for choosing ‘Blue 28’ included comments such as sadness and it is possible that they were linking their choice to colloquial expressions such as ‘having the blues’. However, ‘Blue 28’ was also favoured by a number of HAD normal individuals raising the possibility of a confounding factor. Manchester is a city with an extremely strong football tradition with the colors of one of its football clubs ‘Manchester City’, being very close to ‘Blue 28’. Consequently, when interpreting the results of mood color choice it is important to take account of local factors that might influence choice as well as the fact that in different cultures, different colors may have different connotations [18,19]. Green was also favoured in adolescents with anxiety and we have previously observed this, although to a lesser extent, in adults with this finding being consistent with an earlier report suggesting that green is associated with the word ‘anxious’ [20].

### Study 2 – Short term mood change in response to differing activities

All three activities designed to improve mood resulted in more pupils choosing a positive color by the afternoon compared to the morning, whereas in controls the reverse happened with more participants choosing a negative color by the afternoon. There was an approximately 17% shift towards positive colors with all three interventions with a significance level of p = 0.047 for sport and p = 0.054 for music. In the Art Group there was a preponderance of participants who initially chose ‘Purple 17’ and it has been claimed that some purples and purple-blues are actually positive colors [21]. In our original validation study we did find that some lighter shades of purple were positive but ‘Purple 17’ was consistently found to be a negative shade [3]. We therefore think that it is reasonable to still conclude that there was a shift in color choice from negative to positive even though the numbers were too small to draw a meaningful conclusion.

There is a considerable amount of evidence that activities such as sport and music have a beneficial effect on mood and art has been used as a form of therapy for many years. For instance, exercise has been shown to reduce anxiety and depression as well as being useful in reducing anger and hostility [22-26] and it is likely that these effects are mediated by a variety of neuropeptides [22]. Using brain imaging techniques, music has been shown to activate cortical areas associated with pleasure and emotion [27,28] and has been shown to positively affect mood [29-32] which is also likely to have a neurochemical basis [27]. Although Art Therapy is widely used as a psychotherapeutic modality, for instance, to facilitate self expression [33,34] and coping [35,36] there is surprisingly little evidence on its effect on anxiety and depression although in this study it did appear to have an effect despite significance not being reached.

It might initially seem surprising that short interventions, such as those used in this study, could lead to a change in mood, as reflected in mood color choice, in such a significant proportion of pupils. However, this appears to be a robust finding as not only did this not occur in controls but the pattern was the exact opposite. A possible explanation for this finding is that mood tends to be especially volatile in adolescents and is therefore more easily susceptible to change. Conversely, it might be anticipated that a low mood state may be more stable in adults and it would be of interest to assess whether in such individuals there would be increased resistance to similar short term changes.

## Conclusion

The results of this study indicate that the MCW is a valid instrument for use in adolescents and is sensitive to change. Consequently, even though it is unlikely that it is detecting exactly the same psychological state as a depression questionnaire, it may be a useful rapid screening tool for such purposes in large populations of adolescents where the use of long and sometimes rather intrusive questionnaires would be impractical. Our results also indicate that there might be a small but significant number of pupils with a mood disorder and the use of the MCW instrument is a quick and simple way to identify adolescents who may benefit from further investigation, support or intervention. For instance, it is noteworthy that in a recent study on self-harm in adolescents a link with low mood was identified and the authors emphasised the importance of recognising individuals at risk [37]. In addition, the application of the MCW is not necessarily confined to mood; it could be used for gauging the response to a whole variety of questions which in a school, for instance, could be how students perceive the subjects in their curriculum. Another advantage of this instrument, especially for use in population studies, is that it can easily be converted to an electronic form or even an ‘app’ for a smart phone. It might be considered that the utility of the MCW could be limited by color vision deficiency (color blindness), but this problem only occurs in up to 8% of males and 0.5% of females [38]. However, this potential drawback is less of a problem than “functional illiteracy” which has been estimated to affect 16% of the U.K. population [39] and impairs the ability to understand written medical materials [40] such as psychologically orientated questionnaires.

For the purposes of validation, this study was dependant on us finding some individuals with a low mood state using the HAD Scale. However, unlike questionnaires designed to identify anxiety or depression, the MCW is also capable of detecting a more positive frame of mind. It is therefore encouraging that nearly half the pupils in this school, far from being just neutral, actually chose a positive color to describe their mood.

## Appendix

Appendix 1: Central Manchester University Hospitals NHS Foundation Trust; Manchester Mental Health and Social Care Trust; Salford Primary Care Trust (NHS Salford); Salford Royal NHS Foundation Trust; The Christie NHS Foundation Trust; and University Hospital of South Manchester NHS Foundation Trust.

## Abbreviations

MCW: Manchester Color Wheel; HAD: Hospital Anxiety and Depression Scale.

## Competing interests

The authors declare that they have no competing interests.

## Authors’ contributions

HRC, LM, SO and PJW conceived and designed the study. LKH was responsible for organising the data collection. HRC inputted and undertook the statistical analysis of the data. HRC and PJW drafted the manuscript. LM, SO and LKH reviewed, critiqued and revised the manuscript. All authors have read and approved the final version of the paper.

## Pre-publication history

The pre-publication history for this paper can be accessed here:

http://www.biomedcentral.com/1471-2288/12/136/prepub
